# The influence of acculturation on the risk of stillbirth in migrant women residing in Western Australia

**DOI:** 10.1371/journal.pone.0231106

**Published:** 2020-04-02

**Authors:** Maryam Mozooni, David Brian Preen, Craig Edward Pennell

**Affiliations:** 1 School of Population and Global Health, The University of Western Australia, Perth, WA, Australia; 2 School of Medicine and Public Health, The University of Newcastle, Callaghan, NSW, Australia; Anglia Ruskin University, UNITED KINGDOM

## Abstract

**Objective:**

To investigate the influence of acculturation, demonstrated by age on arrival, length of residence, interpreter use and having an Australian-born partner, on disparities observed in the risk of stillbirth between migrant and Australian-born populations in Western Australia (WA).

**Methods:**

A retrospective cohort study using linked administrative health data for all non-Indigenous births in WA from 2005–2013 was performed. Logistic regression analysis was used to estimate odds ratios (OR) and 95% confidence intervals (CI). Adjusted odds ratios (aOR) for stillbirth in migrants from six ethnicities of white, Asian, Indian, African, Māori, and ‘other’, with different levels of acculturation, were compared with Australian-born women using multivariable logistic regression analysis and marital status, maternal age group, socioeconomic status, parity, plurality, previous stillbirth, any medical conditions, any pregnancy complications, sex of baby, and smoking during pregnancy as the covariates.

**Results:**

From all births studied, 172,571 (66%) were to Australian-born women and 88,395 (34%) to migrant women. Women from African, Indian and Asian backgrounds who gave birth in the first two years after arrival in Australia experienced the highest risk of stillbirth (aOR 3.32; 95% CI 1.70–6.47, aOR 2.71; 95% CI 1.58–4.65, aOR 1.93; 95% CI 1.21–3.05 respectively) compared with Australian-born women. This association attenuated with an increase in the length of residence in Asian and Indian women, but the risk of stillbirth remained elevated in African women after five years of residence (aOR 1.96 [1.10–3.49]). Interpreter use and an Australian-born partner were associated with 56% and 20% lower odds of stillbirth in migrants (p<0.05), respectively.

**Conclusions:**

Acculturation is a multidimensional process and may lower the risk of stillbirth through better communication and service utilisation and elevate such risk through increase in prevalence of smoking in pregnancy; the final outcome depends on how these factors are in play in a population. It is noteworthy that in women of African background risk of stillbirth remained elevated for longer periods after immigrating to Australia extending beyond five years. For migrants from Asian and Indian backgrounds, access to services, in the first two years of residence, may be more relevant. Enhanced understanding of barriers to accessing health services and factors influencing and influenced by acculturation may help developing interventions to reduce the burden of stillbirth in identified at-risk groups.

## Introduction

The majority of international migrants live in high-income countries (HICs) [1]. According to the United Nations’ International Migration Report 2015, 28% of the total population in Australia were migrants, representing a greater proportion than in countries such as the United States (US) (14%), Canada (22%), and New Zealand (23%) [1]. Migrants are considered a vulnerable population given the social, economic, environmental, and occupational disadvantages to which they are often exposed; yet, in some settings, they are healthier than the host country’s population [2]. However, research also shows that foreign-born women and ethnic minorities in the United States (US) [3], Europe [4,5], and Australia [6,7] have a higher risk of stillbirth compared with their native-born counterparts. The fetal mortality rate for non-Hispanic black women in the US was more than twice the rate for non-Hispanic white women [3], the risk of stillbirth among Turkish mothers in Europe was 1.6-times that of the native-born Europeans [5], and for African women more than twice that of Australian-born women [6].

Stillbirth profoundly impacts families, society, and healthcare systems [8] and efforts to reduce the stillbirth rate have recently gained momentum globally [9–11]. For migrant women, unfamiliarity with new healthcare systems, language barriers, sociocultural factors, and health habits may contribute to the risk of stillbirth [6,12]. Acculturation is the cultural, psychological and behavioural changes experienced by migrants as a result of interaction with the host community over time [13]. Variables such as country of birth, ethnic identification, proficiency in language, and length of residence in the new country, have been used as proxies to measure acculturation in relation to health outcomes among migrants in other settings [14–17]. Evidence from the US and Europe suggests that acculturation, perhaps through lifestyle modification [14,15], may improve birthweight and gestational-age in some migrant populations [16] but can negatively impact these outcomes in others [17]. Further, a lower risk of stillbirth was reported in migrant women whose baby was registered with a Norwegian-born father compared to those with foreign-born fathers, suggesting a potential benefit from acculturation for stillbirth risk [18]. Similar research is limited in Australia and the impact of acculturation on stillbirth risk is poorly understood; we have previously shown that the migrant population, compared with the Australian-born population, utilise healthcare services differently. Late commencement of antenatal-care visits and lack of access to or uptake of doctor-provided intrapartum-care as well as absence of private health insurance emerged as underlying factors for increased risk of stillbirth in specific ethnic groups in Western Australia (WA) [19]. However, it is not clear whether the risk of stillbirth persists the longer migrants live and interact with the community in Australia.

We investigated the influence of acculturation on disparities observed in the risk of stillbirth between migrants from diverse ethnic backgrounds and Australian-born populations in WA from 2005 to 2013. Specifically, the effect of length of residence, age on arrival, interpreter use and having an Australian-born partner were explored as proxies for acculturation.

## Methods

### Study design and participants

De-identified administrative health data for all non-Indigenous births in WA, from 1 January 2005 to 31 December 2013, linked through the WA Data Linkage System (WADLS) of WA Department of Health [20,21], were utilised for this retrospective cohort study.

### Data sources

WADLS was established in 1995, mainly for population health research purposes as a collaborative work between the WA Department of Health and researchers. It has successfully linked data dating back to the 1970s and has provided support to more than 400 studies just in its first ten years of operation [21]. The linkage procedures used are widely known as “best practice” [21,22]. Using numerous automated and manual sub-processes specifically designed to reduce the likelihood of errors, WADLS prides itself on the highest quality of the linkages it produces [23,24].

For this study a range of statutory data collections was used including the Midwives’ Notification System (MNS), the Hospital Morbidity Data Collection (HMDC), Birth and Death Registrations and the WA Registry of Developmental Anomalies (WARDA). Genealogical linkage through the Family Connections Linkage Facility of the WADLS [25] was used to link mothers with child records. The details of the datasets used were described elsewhere [6,19].

Data from MNS, Birth Registration and HMDC records on the country of birth and ethnicity of mothers and their migrant status (migrant = overseas-born) were available for 99.99% of women through cross-source checking. The migrant population was further stratified by self-reported maternal ethnicity (from MNS) as white (Caucasian), Asian, Indian, African, Māori, and ‘other’ which was available for the entire population of study. It is worth noting that in Australia, women born in India or those from an Indian background born elsewhere are categorised separately and are not classified within the Asian group for which the majority were born in China, Vietnam, Malaysia, or Indonesia [6].

### Exposure and outcome variables

Stillbirth (defined as the death of a baby of at least 20 completed weeks of gestation, or 400 grams or more birthweight, before the complete expulsion or extraction from the mother) [26] was the outcome of interest and was recorded in the status of the baby at birth (MNS data). Terminations of pregnancy, identified and ascertained through WARDA and Death Registrations data, were excluded (n = 433).

We used length of residence, age on arrival, interpreter use and having an Australian-born partner as proxies for acculturation [27–29]. Interpreter-used (yes/no) was available (from HMDC) for all births in hospital (and 99.0% of total births in WA). Partner’s country of birth, through birth registration, was available for 96.8% of women. Length of residence (years) was calculated by subtracting year arrived in Australia (from Birth registration) from the year of birth of the baby (from MNS) with 5.7% missing data (on mother’s arrival year). Age on arrival was calculated by subtracting women’s length of residence from their age at time of birth (from MNS). Arriving as an adult, utilising interpreter service, not having an Australian-born partner, and residing in Australia for less than five years are considered the indicators of being less acculturated [27–29].

Private-health-insurance-status (yes/no) was also available for all births in hospital (from HMDC). Index of Relative Socioeconomic Disadvantage (IRSD), an area-based measure of socioeconomic status developed by the Australian Bureau of Statistics summarising several disadvantage measures including low income, low education, high unemployment and unskilled occupations [30], was available for 96.7% of women through multiple datasets.

Missing data were categorized as a separate subgroup to retain all cases in the analysis.

### Statistical analysis

Demographic and obstetric characteristics of the study groups were tabulated. The cumulative incidence rate of stillbirth was calculated over the period of study, stratified by ethnicity with denominators determined by 10,000 total birth (live and stillbirth). Univariate logistic regression was initially used to examine the crude association of stillbirth with acculturative factors (interpreter use, length of residence, age on arrival, and Australian-born partner) at the p<0.05 level. To determine the adjusted odds ratios (aORs) and 95% confidence interval (CI) of stillbirth, multivariable logistic regression analysis was performed for each specific ethnicity with Australian-born as the reference group, adjusting for marital status, maternal age group, socioeconomic status, parity, plurality, previous stillbirth, any medical conditions, any pregnancy complications, sex of baby and smoking during pregnancy. Where stratifying the analysis by specific ethnic groups was not possible due to small numbers, the whole population of migrants were compared with the Australian-born group but ethnicity was added to the multivariable regression model as a co-variable to control for the effect of ethnicity.

Exploratory analysis was undertaken by combining Asian and Indian population and adding private health insurance, as a measure of access to services, to the multivariable model.

Sensitivity analysis was performed by excluding stillbirths with major anomalies.

All analyses were performed using Stata (version 13·1; StataCorp LP, College Station, Texas).

Ethics approval for this study was granted by the Human Research Ethics Committee of the WA Department of Health (reference, 2015/23). Written consent from participants was not required to conduct the study due to the use of non-identifiable routinely collected linked administrative health data for the whole population.

## Results

Demographic data for the study population are presented in Table 1. From 260,997 total non-Indigenous births, 172,571 births (66%) were to Australian-born women and 88,395 births (34%) to migrant women. Migrant women were, on average, slightly older than Australian-born women (the mean age 30.9 years vs 29.5 years, respectively), and more likely to be married (91.7% vs 88.0%), non-smokers (7.1% vs 14.0%) and nulliparous (44.0% vs 42.6%), but less likely to have private health insurance (32.1% vs 43.1%). In contrast, the proportion of migrant women categorised as the most socioeconomically disadvantaged (using IRSD quintiles) was 12% less than Australian-born women (p<0.001). Majority of migrants arrived in Australia as an adult, aged ≥18 years old, and almost 12% of the whole population of migrant women gave birth before completing two years of residence in Australia (Table 1). Among the migrant population, 30.0% had an Australian-born partner with African and Indian women having the lowest (4.5% and 4.8%, respectively) and Māori and Asian women having the highest (23.4% and 19.7%, respectively) percentage of Australian-born partners after white migrants (41.6%). African women had the highest proportion of interpreter utilisation (15.7%), followed by ‘other’ (10.4%) and Asian (9.8%) women, while no woman from Māori background utilised such services.

**Table 1:** Characteristics of the population of the study.

Characteristics	Australian-born women	Migrant women							All women
		White	Asian	Indian	African	Māori	Other	All Migrants	
**Total births**	172 571	48 546	18 212	5503	4155	2941	9038	88 395	260 997
**Stillbirth**	812(0.5%)	231(0.5%)	95(0.5%)	39(0.7%)	51(1.2%)	18(0.6%)	66(0.7%)	500(0.6%)	1313(0.5%)
**Marital status**									
Never married	18 016(10.4%)	3026(6.2%)	701(3.9%)	99(1.8%)	543(13.1%)	570(19.4%)	611(6.8%)	5550(6.3%)	23 568(9.0%)
Divorced/separated	1554(0.9%)	360(0.7%)	160(0.9%)	17(0.3%)	109(2.6%)	33(1.1%)	132(1.5%)	811(0.9%)	2366(0.9%)
Married/de facto	151 831(88.0%)	44 693(92.1%)	17 107(93.9%)	5327(96.8%)	3449(83.0%)	2268(77.1%)	8214(90.9%)	81058(91.7%)	232 917(89.2%)
Other	1170(0.7%)	467(1.0%)	244(1.3%)	60(1.1%)	54(1.3%)	70(2.4%)	81(0.9%)	976(1.1%)	2146(0.8%)
**Australian-born Partner**	118401(71.8)	19503(41.6%)	3 535(19.7%)	264(4.8%)	178(4.5%)	633 (23.4%)	1540(17.6%)	25652(30.0%)	144 053(57.5%)
**Age on arrival**									
<18 years old	NA	20273(43.9%)	4233(24.7%)	433(8.4%)	604(16.1%)	1016(36.8%)	2068(24.8%)	28627(34.4%)	NA
18 or older	NA	25909(56.1%)	12904(75.3%)	4741(91.6%)	3140(83.9%)	1749(63.3%)	6257(75.2%)	54700(65.6%)	NA
**Length of residence**									
<2years	NA	4048(8.3%)	2361(13.0%)	1299(23.6%)	729(17.6%)	527(17.9%)	1571(17.4%)	10535(11.9%)	10537(4.0%)
2–5 years	NA	11380(23.4%)	5399(29.7%)	2613(47.5%)	1595(38.4%)	857(29.0%)	2787(30.8%)	24631(27.9%)	24634(9.4%)
>5 years	NA	30 769(63.4%)	9384(51.5%)	1262(22.9%)	1419(34.2%)	1382(47.0%)	3968(43.9%)	48184(54.5%)	48188 (18.5%)
Unknown	NA	2 349(4.8%)	1068(5.9%)	329(6.0%)	412(9.9%)	175(6.0%)	712(7.9%)	5045(5.7%)	5082(2.0%)
**Maternal age (years)**									
Mean (SD)	29.5(5.6)	31.5(5.3)	31.2(4.9)	29.5(4.4)	28.8(5.7)	26.8(6.0)	29.9(5.6)	30.9(5.4)	30.0(5.6)
< 20	7474(4.3%)	724(1.5%)	131(0.7%)	17(0.3%)	197(4.7%)	300(10.2%)	200(2.2%)	1569(1.8%)	9045(3.5%)
20–24	27 516(16.0%)	4364(9.0%)	1401(7.7%)	638(11.6%)	826(19.9%)	889(30.2%)	1477(16.3%)	9595(10.9%)	37 115(14.2%)
25–29	49 076(28.4%)	11423(23.5%)	5208(28.6%)	2288(41.6%)	1254(30.2%)	786(26.7%)	2631(29.1%)	23590(26.7%)	72 673(27.8%)
30–34	54 744(31.7%)	17464(36.0%)	6937(38.1%)	1869(34.0%)	1169(28.1%)	599(20.4%)	2714(30.0%)	30752(34.8%)	85 510(32.8%)
35–39	28 412(16.5%)	11716(24.1%)	3713(20.4%)	581(10.6%)	586(14.1%)	290(9.9%)	1612(17.8%)	18498(20.9%)	46 914(18.0%)
40–44	5159(3.0%)	2703(5.6%)	785(4.3%)	103(1.9%)	112(2.7%)	77(2.6%)	389(4.3%)	4169(4.7%)	9328(3.6%)
> 44	190(0.1%)	152(0.3%)	37(0.2%)	<10(0.1%)	11(0.3%)	0(0.0%)	15(0.2%)	222(0.3%)	412(0.2%)
**Smoked in pregnancy**	24097(14.0%)	4161(8.6%)	317(1.7%)	40(0.7%)	78(1.9%)	1152(39.2%)	494(5.5%)	6242(7.1%)	30 342(11.6%)
**Interpreter service utilised**	19(0·0%)	306(0·6%)	1779(9·8%)	232(4·2%)	651(15·7%)	0(0·0)	931(10·4%)	3896(4.5%)	3918(1·5%)
Socioeconomically* **most disadvantaged**	40521(23·5%)	6547(13·5%)	2181(12·0%)	663(12·1%)	513(12·4%)	800(27·2%)	1276(14·1%)	11980(13.6%)	52504(20·1%)
**Private health insurance**	73774(43·1%)	19247(40·2%)	5495(30·2%)	1379(25·0%)	380(9.2%)	153(5·3%)	1471(16·3%)	59374(32.1%)	101902(39·4)
**Parity**									
Nulliparous	73 456(42.6%)	21 205(43.7%)	8759(48.1%)	3204(58.2%)	1217(29.3%)	955(32.5%)	3532(39.1%)	38872(44.0%)	112 340(43.0%)
Primiparous	60 403(35.1%)	17 243(35.5%)	6485(35.6%)	1817(33.0%)	1113(26.8%)	792(26.9%)	2695(29.8%)	30145(34.1%)	90 561(34.7%)
Multiparous	38 712(22.5%)	10 098(20.8%)	2968(16.3%)	482(8.8%)	1825(43.9%)	1194(40.6%)	2811(31.1%)	19378(21.9%)	58 096(22.3%)
**Any medical condition**	60 602(35.4%)	14 748(30.8%)	4 889(26.9%)	2095(38.0%)	1567(37.9%)	993(34.2%)	3299(36.8%)	27590(31.5%)	60603(35.3%)
**Previous stillbirth**	2011(1.2%)	575(1.2%)	179(1.0%)	73(1.3%)	149(3.6%)	53(1.8%)	182(2.0%)	1211(1.4%)	3222(1.3%)
**Any pregnancy complication**	57596(33.4%)	15330(31.6%)	6489(35.6%)	2162(39.3%)	1339(32.2%)	925(31.5%)	3214(35.6%)	29459(33.3%)	87 059(33.4%)
**Plurality**									
Singleton	167 481(97.1%)	47 075(97.0%)	17 822(97.9%)	5389(97.9%)	4031(97.0%)	2883(98.0%)	8725(96.5%)	85925(97.2%)	253435(97.1%)
Multiple	5090(3.0%)	1471(3.0%)	390(2.1%)	114(2.1%)	124(3.0%)	58(2.0%)	313(3.5%)	2470(2.8%)	7544(2.9%)

*The socioeconomically most disadvantaged group is comprised of the bottom 20% of IRSD.

The period “<2 years of residence” had the highest odds of stillbirth in migrant women (OR 1.35; 95% CI 1.05–1.74) compared to the Australian-born women (Table 2). The odds were 14% higher among migrant women who immigrated as an adult, compared with Australian-born population, although it did not reach statistical significance (OR 1.14, 95% CI 1.00–1.30). Compared to Australian-born women, the non-white-non-Māori migrant women who did not utilise an interpreter were at higher odds (OR 1.55; 95% CI 1.34–1.79) and those who did have an interpreter had a lower risk (OR 0.47; 95% CI 0.24–0.95) of stillbirth (Table 2).

**Table 2:** Absolute numbers, rates, and unadjusted odds ratios of stillbirth for migrants, stratified by acculturative factors, compared with the Australian-born population.

Acculturative factor	Stillbirth		
	N	Rate (per 10,000 births)	OR (95% CI)
**Australian-born (Reference)**	812	47	1.00
**Overseas-born**	500	57	1.20* (1.08–1.35)
**Interpreter use**			
Non-white-non-Māori migrant with interpreter	<10	22	0.47* (0.24–0.95)
Non-white-non-Māori migrant without interpreter	240	72	1.55* (1.34–1.79)
**Length of residence**			
<2 y	66	63	1.35* (1.05–1.74)
2–5 y	134	55	1.17 (0.97–1.41)
>5 y	220	46	0.98 (0.85–1.14)
**Age on arrival**			
<18 years old	133	46	0.98 (0.82–1.18)
≥18 years old	293	54	1.14 (1.00–1.30)
**Australian-born partner**			
Migrant with Australian-born partner	118	46	0.97 (0.80–1.17)
Migrant with overseas-born partner	336	56	1.30* (1.15–1.47)

* P<0.05

When stratified by ethnicity, the greatest association with lack of utilisation of interpreter services was seen in African (aOR 3.16; 95% CI 2.34–4.26), followed by women from ‘other’ (aOR 1.65; 95% CI 1.27–2.14) and Indian (aOR 1.58; 95% CI 1.13–2.22) ethnic backgrounds.

**Fig 1:**
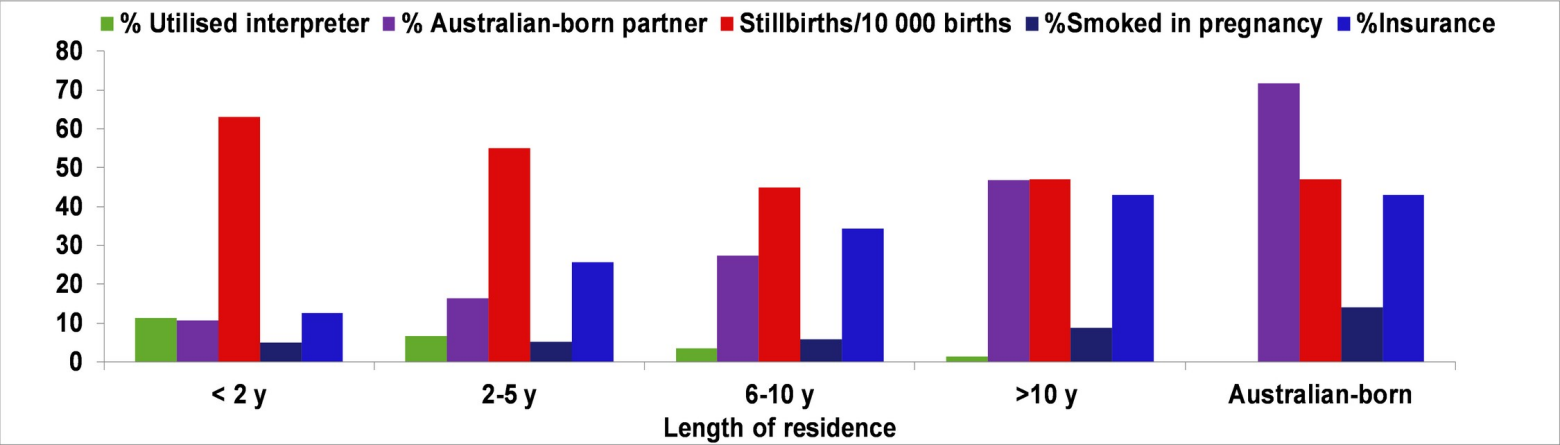
Length of residence in relation to utilisation of interpreter, having an Australian-born partner, smoking in pregnancy and rate of stillbirth in the migrant population of Western Australia (2005–2013).

The cumulative rate of stillbirth in migrant women steadily decreased reaching the same rate as Australian born women when the length of residence was greater than 10 years (Fig 1). Parallel to that, the proportion of smoking in pregnancy, having an Australian-born partner, as well as private health insurance, increased among migrant women (Fig 1).

When migrant population was stratified by ethnicity, length of residence was not associated with stillbirth in Māori or white migrant women compared to Australian-born women; however, women from Asian and Indian backgrounds had significantly increased odds of stillbirth in their first two years of residence in Australia (aOR 1.93; 95% CI 1.21–3.05, aOR 2.71; 95% CI 1.58–4.65, respectively) which resolved with longer periods of residence. Women from African backgrounds had more than three-times higher odds of stillbirth than their Australian-born counterparts (aOR 3.32; 95% CI 1.70–6.47) in their first two years of residence. Although the risk of stillbirth decreased with longer residence periods in these women, it remained significantly higher than Australian-born women after five years of residing in Australia (aOR 1.96; 95% CI 1.10–3.49). This risk persisted after adjusting for other stillbirth risk factors (Table 3).

**Table 3:** Length of residence and the odds of stillbirth in migrants from specific ethnic backgrounds compared to the Australian-born population.

Study population (N)			Length of residence		
			<2 years	2–5 years	>5 years
**Australian-born (Reference)**			1.00	1.00	1.00
**Migrant**					
White	OR (95% CI)		0.73 (0.43–1.25)	0.99 (0.75–1.31)	0.94 (0.78–1.13)
	aOR^†^ (95% CI)		0.88 (0.52–1.50)	1.20 (0.90–1.59)	1.02 (0.84–1.23)
Asian	OR (95% CI)		1.71* **(1.09–2.71)**	0.90 (0.60–1.37)	1.00 (0.73–1.35)
	aOR^†^ (95% CI)		1.93* **(1.21–3.05)**	1.01 (0.66–1.53)	1.06 (0.77–1.44)
Indian	OR (95% CI)		2.30* **(1.35–3.91)**	1.22 (0.73–2.04)	0.67 (0.25–1.79)
	aOR^†^ (95% CI)		2.71* **(1.58–4.65)**	1.38 (0.82–2.32)	0.67 (0.25–1.80)
African	OR (95% CI)		2.64* **(1.36–5.11)**	2.41* **(1.51–3.85)**	1.80* **(1.01–3.19)**
	aOR^†^ (95% CI)		3.32* **(1.70–6.47)**	2.77* **(1.70–4.52)**	1.96* **(1.10–3.49)**
Māori	OR (95% CI)		0.80 (0.20–3.22)	1.74 (0.82–3.66)	1.07 (0.51–2.26)
	aOR^†^ (95% CI)		0.75 (0.19–3.03)	1.71 (0.70–3.62)	1.07 (0.51–2.28)
Other	OR (95% CI)		1.08 (0.54–2.17)	1.53 (0.98–2.38)	1.07 (0.69–1.67)
	aOR^†^ (95% CI)		1.23 (0.61–2.49)	1.63* **(1.04–2.56)**	1.08 (0.74–1.80)

^**†**^aOR: Adjusted for marital status, maternal age group, socioeconomic status, parity, plurality, previous stillbirth, medical conditions, pregnancy complications, sex of baby and smoking during pregnancy.

*P<0.05

**Fig 2:**
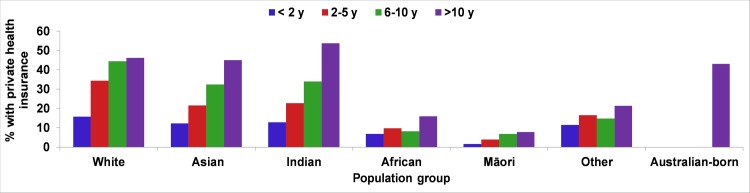
Length of residence in Australia and percentage of having private health insurance for migrant women from specific ethnic backgrounds delivered in WA (2005–2013).

The increase in proportion of the migrant population with private health insurance was most evident among women from white (15.8% in <2 years of residence to 34.4% in 2–5 years of residence), Asian (12.2% in <2 years of residence to 21.6% in 2–5 years of residence) and Indian (12.8% in <2 years of residence to 22.7% in 2–5 years of residence) backgrounds (Fig 2). It is worth noting that migrant women from these ethnic backgrounds, who lived in Australia for >10 years, had a higher percentage of private health insurance than Australian-born women (46.2%, 45.1%, 53.8%, respectively).

In further exploratory analysis, including ‘having private health insurance’ in the multivariable model resulted in 33% reduction in the odds of stillbirth in Asian and Indian (combined) women with less than two years length of residence (from aOR 2.18; 95% CI 1.52–3.10 to aOR 1.85; 95% CI 1.29–2.65).

**Fig 3:**
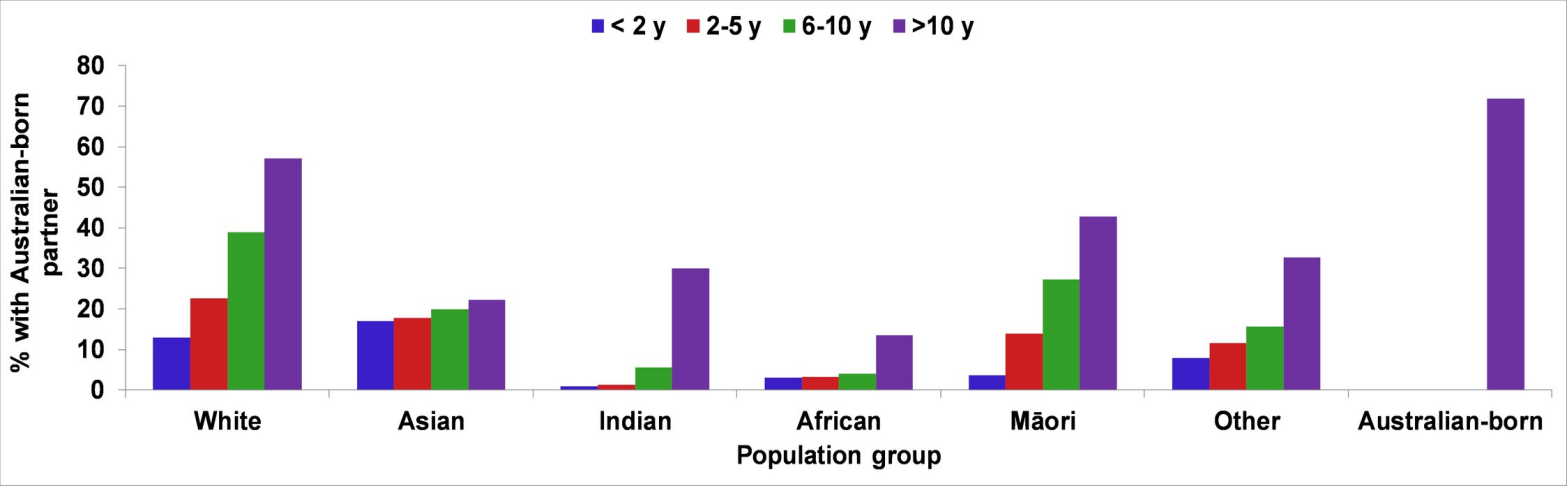
Length of residence in Australia and percentage of having an Australian-born partner for migrant women from specific ethnic backgrounds delivered in WA (2005–2013).

The proportion of the population with an Australian-born partner is illustrated in Fig 3 for each specific ethnic group. Overall, 71.8% of Australian-born and 30% of migrant women had an Australian-born partner. The proportion of migrant women with an Australian-born partner increased with a longer length of residence for all migrant groups, however, the rate varied for each specific ethnicity with Indian women having the highest surge after ten years of residing in Australia.

**Fig 4:**
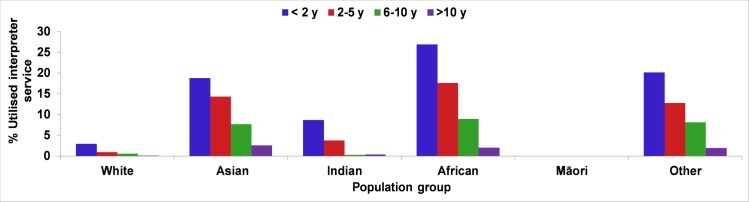
Length of residence in Australia and the percentage of utilisation of interpreter for migrant women from specific ethnic backgrounds delivered in WA (2005–2013).

The proportion of interpreter utilisation was the highest in the first two years of residence in Australia and gradually decreased with longer length of residence, albeit at various rates among different ethnic groups of migrants (Fig 4).

### The overall level of acculturation

Migrant women with an overseas-born partner who did not utilise an interpreter were the most at-risk group among new migrants residing in Australia for less than five years. While their migrant counterparts who utilised an interpreter had 62% lower odds of stillbirth (aOR 0.38; 95% CI 0.15–0.95), these women had a 31% increased odds of stillbirth (aOR 1.31; 95% CI 1.03–1.65), compared to Australian-born women. No significant difference was observed in those with a length of residence of >5 years. Non-white-non-Māori migrant women with an overseas-born partner who did not utilise an interpreter were particularly at risk with 86% increased odds of stillbirth (aOR 1.86; 95% CI 1.50–2.32) compared with Australian-born women.

Sensitivity analyses, excluding stillbirths with major congenital anomalies, did not affect the findings.

## Discussion

We found that longer length of residence, utilising an interpreter, and having an Australian-born partner were all associated with a lower risk of stillbirth in migrants. Further, we showed that migrant women with an overseas-born partner, who did not utilise an interpreter, were the most at-risk group among migrants residing in Australia <5 years. Giving birth in the first two years after arrival in Australia had the highest risk of stillbirth for migrant women from African, Asian and Indian backgrounds. However, the increased risk disappeared beyond the first two years of residence among women from Asian and Indian backgrounds.

A longer length of residence in Australia was associated with a lower risk of stillbirth in migrants in this study, consistent with a previous nationwide study in Sweden where the risk of stillbirth was higher in foreign-born women with <5 years of residence [31]. In contrast, a recently published nationwide population-based study from Norway did not find an association between the length of residence and stillbirth in immigrant women [18]. The discrepancy in these two Nordic studies, with seemingly comparable populations and health system, is probably due to the differences in the definition of outcome or lack of adjustments for potential confounders such as smoking and body mass index in the Norwegian study. Sweden defines stillbirth as the death of a baby of at least 28 weeks of gestation while Norway uses a lower limit of 22 weeks gestation. Smoking is a well-established risk factor for stillbirth [32]. In our study, the more acculturated women (as indicated by having an Australian-born partner and longer length of residence) were more likely to smoke while pregnant. Elevated rates of smoking in pregnant Hispanic women with a higher level of acculturation have also been reported in the US [33] and among Turkish women residing in Europe [14]. Given that smoking is a risk factor for stillbirth, the protective effect of longer length of residence on stillbirth may have been counteracted and underestimated due to the simultaneous increase in the prevalence of smoking in more acculturated women.

Of note, adjusting for private health insurance status reduced the odds of stillbirth by 33% in Asian and Indian women resided in Australia for <2 years in our study. This may suggest that rather than the acculturative factors, unfamiliarity with the health system or other barriers to access may influence the risk of stillbirth in newly arrived migrants from these ethnic backgrounds. In Australia, despite availability of a universal health insurance scheme covering all Australian citizens and permanent residents (Medicare), depending on type of visa (Humanitarian visas are excluded), a two to four years waiting period is applied before immigrants become eligible to benefit from some health and social security services including Newstart Allowance and Healthcare Concession Card [34]. Whether this operates as a barrier to service utilisation and is associated with the observed disparities in the rate of stillbirth among migrant women who gave birth before completed two years of residence needs further investigation.

Effective communication is vital for navigating the health system and for optimal care. Language discordance has previously been highlighted as a barrier to access health services for migrants and refugees in Australian and to compromise the quality of care [35–37]. In our study, migrant women who utilised an interpreter had a significantly lower risk of stillbirth compared to those who did not. Given the majority of migrants who utilised an interpreter were from regions with higher rates of stillbirth than Australia and had a residence length of <5 years in Australia, this may be evidence of a “healthy migrant paradox” [38]. This implies observing better health outcomes among newly arrived migrants than the host population, despite the disadvantages migrants encounter, which slowly converges to the host population levels over time [38]. Not utilising an interpreter was a particularly strong risk factor for non-white-non-Māori migrant women who had an overseas-born partner and delivered during the first five years of residing in Australia, suggesting lower levels of acculturation. Anecdotal evidence and qualitative studies would suggest that a reasonable proportion of women who did not utilise interpreter service were not competent in the language. Previous reports from Australia and Europe indicated that despite the difficulty in communication, migrant women from some ethnic backgrounds may not request an interpreter or their partners may insist on acting as the translator which can compromise the care received [37,39–41]. Despite the availability of a unique fee-free rapid-access telephone interpreter service (the Doctors Priority Line) in Australia, it was estimated that for less than 1% of private general practice consultations for patients with poor English proficiency, this service was used [41]. Further, qualitative studies have shown doctors to indicate a tendency for over-investigation and acting on the results rather than organising an interpreter and attending to patients’ symptoms [37]. Thus, our findings may indicate a lack of communication and mutual understanding between pregnant women and clinicians in those who did not utilise the interpreter service if the interpreter was required but was not requested or offered. Utilising an interpreter in our data may also be an indicator that a culturally-sensitive healthcare plan was in effect. Mutual accommodation is required for healthy and successful integration of migrants; *adopting* the basic values of the host country by migrants as well as *adapting* the healthcare and education systems to appropriately meet the needs of all population groups [13]. Hence, the lower rate of stillbirth in migrants utilised interpreter services may signify the success of a culturally-sensitive healthcare practice.

Our data suggest a slower acculturation rate in some ethnic groups, particularly those of African descent. The rate of stillbirth in African migrant women decreased with longer duration of residence but remained significantly higher than Australian-born women. Slower acculturation may be a sign of marginalisation or segregation, instead of a healthy integration, potentially due to discrimination, which can also be detrimental to the wellbeing of individuals and the community [13]. This can be more prevalent for those whose physical features (e.g. skin colour or clothing) set them apart from the majority population [13]. A negative association between length of residence and level of stress has been reported in African migrants in the US [42]. Among Somali refugees residing in the US, interpreter service use increased with longer length of residence while lower rates of interpreter utilisation was also associated with poorer birth outcomes [43]. In our population, African women had the highest utilisation of interpreters among all migrant ethnic groups. Despite this, our findings suggest their ‘need’ for interpreters may be higher than currently being met and captured by our data. Addressing under-utilisation of interpreters may be a simple strategy to address the increased risk of stillbirth in this population. This finding also warrants further investigation whether racial discrimination, a risk factor for adverse birth outcomes [44], also plays a role or not.

Consistent with the Norwegian study [18], having an Australian-born partner reduced the risk of stillbirth in migrant women in WA. Intermarriage is considered one of the most powerful indicators of integration [45]. Having an Australian-born partner may result in more interaction within the community, competency in English, as well as familiarity with the health system when navigating pregnancy care [18,46].

In conclusion, migrant populations are diverse and the processes of immigration and acculturation are complex. Leaving family and friends behind and lack of support in the new country, isolation and experience of discrimination may impact the physical and mental health of migrants [42] and consequently be detrimental to the health of their babies. Thus, understanding the effect of migration and acculturation on health of immigrants and their babies is vital to identify disparities and to target at-risk groups with a culturally responsive healthcare system and ethnic-specific preventive strategies. Furthermore, investigating the social, cultural, behavioural and other determinants of lower/slower acculturation is warranted to identify opportunities for intervention and prevention of marginalisation.

### Strength and limitations

We utilised de-identified linked administrative health data in this study to investigate stillbirth in a large population of all migrant women. Such method reduced the risk of selection, participation and recall biases and enhanced accuracy through cross-source checking of data from multiple databases, consequently strengthened the reliability of findings; however, has limitations as well due to the extent of variables available or classification of variables, thus some misclassification towards the null may exist. Residual confounding due to the covariates not recorded in the datasets (eg, maternal body mass index) [47], despite controlling for a range of potential risk factors, may remain. Also, the duration of residence may result in different levels of integration and acculturation whether the migrant arrived as an adult or as a child [48]. However, in our study, the majority of non-white-non-Māori migrants (75.2%-91.6%) had arrived in Australia as an adult and the association of this factor with stillbirth was not statistically significant. Further, we assessed acculturation in a multi-dimensional manner by considering additional acculturative factors such as interpreter use and partner’s country of birth. Given the prevalence of stillbirth in Australia, a very large population of participants is required to explore the influence of migration, ethnicity, and acculturation on the risk of stillbirth. We acknowledge, similar to other population studies, we have investigated the effect of acculturation using proxy measures described in detail in this paper. Although an in-depth interview with participants may provide a thorough and more accurate understanding of psychological, cultural and behavioural changes they experience, given the sample size required for such a study as well as other considerations necessary to interview bereaved parents from diverse backgrounds, this would not be practically feasible. Thus, a population-based linked-data study, such as that undertaken here, is the only feasible method for investigation while still provides useful insight into the influences of these factors on stillbirth. Nevertheless, population data collections may be strengthened by linking census data with additional acculturation-related information, such as the language spoken at home or sociocultural preferences or shift of them over time.

### Implications and generalisability

Acculturation may elevate the risk of stillbirth by increasing the prevalence of unhealthy habits, such as smoking, and decrease the risk through empowering migrants for better utilisation of health services and effective communication. Thus, the resultant outcome will depend on how these underlying factors interplay in populations over time. According to our findings and previous studies in other settings, acculturation is emerging as an important factor to consider when providing health care to immigrant populations. Therefore, findings of this study have important implications for policy and practice; a national educational program to familiarise migrants with the health system, their risks, rights and entitlements to healthcare programs, before, during and after pregnancy, as well as a culturally-responsive healthcare system to improve access to and utilisation of interpreter and other services, especially in recently arrived migrants that are at greater risk, may help reduce the rate of stillbirth. It is particularly crucial to investigate and address the underlying mechanisms for the increased risk of stillbirth in the first two years after the arrival in Australia. Until then, pregnant women from non-white non-Māori ethnic backgrounds with a length of residence <2 years may benefit from being treated as high-risk for stillbirth.

Our findings can be generalized to other populations hosting migrants from similar ethnic backgrounds in Australia and New Zealand, European countries and Canada; nevertheless, the interpretation should be with caution due to factors such as different immigration laws, healthcare systems, cultures and lifestyle norms in host countries.

## Supporting information

S1 Data (DOCX)
